# Liprin-α1 modulates cancer cell signaling by transmembrane protein CD82 in adhesive membrane domains linked to cytoskeleton

**DOI:** 10.1186/s12964-018-0253-y

**Published:** 2018-07-13

**Authors:** Henna Pehkonen, Mira Lento, Pernilla von Nandelstadh, Artemis Filippou, Reidar Grénman, Kaisa Lehti, Outi Monni

**Affiliations:** 1 Research Programs Unit, Genome-Scale Biology Program and Medicum, Biochemistry and Developmental Biology, University of Helsinki, Helsinki, Finland; 2 Department of Otorhinolaryngology, Head and Neck Surgery, University of Turku and Turku University Hospital, Turku, Finland; 3 Department of Microbiology, Tumor and Cell Biology (MTC), Karolinska Institutet, Stockholm, Sweden

**Keywords:** Liprin-α1, PPFIA1, CD82, Head and neck cancer, Breast cancer, Invasion, RNA sequencing, Three-dimensional cell culture

## Abstract

**Background:**

*PPFIA1* is located at the 11q13 region commonly amplified in cancer. The protein liprin-α1 encoded by *PPF1A1* contributes to the adhesive and invasive structures of cytoskeletal elements and is located at the invadosomes in cancer cells. However, the precise mechanism of liprin-α1 function in cancer progression has remained elusive.

**Methods:**

Invasion regulating activity of liprin-α1 was examined by analyzing the functions of squamous cell carcinoma of head and neck (HNSCC) cell lines in three-dimensional collagen I after RNAi mediated gene knockdown. Transcriptome profiling and Gene Set Enrichment Analysis from HNSCC and breast cancer cells were used to identify expression changes relevant to specific cellular localizations, biological processes and signaling pathways after *PPFIA1* knockdown. The significance of the results was assessed by relevant statistical methods (Wald and Benjamini-Hochberg). Localization of proteins associated to liprin-α1 was studied by immunofluorescence in 2D and 3D conditions. The association of *PPFIA1* amplification to HNSCC patient survival was explored using The Cancer Genome Atlas data.

**Results:**

In this study, we show that liprin-α1 regulates biological processes related to membrane microdomains in breast carcinoma, as well as protein trafficking, cell-cell and cell-substrate contacts in HNSCC cell lines cultured in three-dimensional matrix. Importantly, we show that in all these cancer cells liprin-α1 knockdown leads to the upregulation of transmembrane protein CD82, which is a suppressor of metastasis in several solid tumors.

**Conclusions:**

Our results provide novel information regarding the function of liprin-α1 in biological processes essential in cancer progression. The results reveal liprin-α1 as a novel regulator of CD82, linking liprin-α1 to the cancer cell invasion and metastasis pathways.

**Electronic supplementary material:**

The online version of this article (10.1186/s12964-018-0253-y) contains supplementary material, which is available to authorized users.

## Background

*PPFIA1* is located at the 11q13 amplification region [1] which is related to poor prognosis of the patients in several cancers, including head and neck squamous cell carcinoma (HNSCC) and breast cancer [2–4]. *PPFIA1* encodes liprin-α1 protein, which is a member of the liprin protein family of tyrosine phosphatase interacting proteins conserved in evolution [5, 6]. Liprin-α proteins have been studied extensively in neurons with reported involvement in synapse functions [7–10].

In addition to the functions in neuronal cells, liprin-α1 has been associated to cancer metastases [11], cell migration and invasive growth [12, 13]. Of note, liprin-α1 affects cancer cell spreading, the distribution of cell surface β1-integrins [14], and regulates cell edge dynamics and focal adhesion assembly in motile epithelial cancer cells via proteins including vimentin, ERC1 (ELKS/RAB6-interacting/CAST family member 1) and β1-integrin [12, 15]. We have recently shown that in non-invasive cancer cells liprin-α1 locates to invadosome structures and promotes growth behavior with limited invasive capacity [12], whereas in invasive and motile cancer cells liprin-α1 is essential for mesenchymal cancer cell invasion and regulation of extracellular matrix degradation [12, 13]. Besides the cancer promoting functions, liprin-α1 has been recently implicated in recycling of active α_5_β_1_ in fibronectin polymerization-dependent vascular morphogenesis [16]. These results suggest several important cellular functions of liprin-α1 in both neuronal and epithelial cancer cells.

In the present study, our aim was to explore the cellular liprin-α1 functions in three-dimensional (3D) collagen I matrix environment, and to identify genes and molecular mechanisms that are involved in liprin-α1 mediated regulation of cell invasive growth. Our results revealed a unique interplay between liprin-α1 and CD82 transmembrane protein in the invasion of HNSCC and breast cancer cells, thus providing mechanistic details of liprin-α1 function in cancer cell progression.

## Methods

### Cell lines and reagents

Two breast cancer cell lines MDA-MB-231 from metastatic breast adenocarcinoma and Hs578T cell line from breast carcinoma (ATCC, American Type Culture Collection, Manassas, MD, USA) were studied. HNSCC cell lines UT-SCC-42A from laryngeal cancer, UT-SCC-42B from corresponding neck metastasis, UT-SCC-19B from laryngeal persistent cancer and UT-SCC-24B from neck metastasis of tongue cancer were derived from clinical samples (Reidar Grénman, Department of Otorhinolaryngology – Head and Neck Surgery, Turku University Hospital, Finland). UT-SCC and MDA-MB-231 cell lines were cultured using Dulbecco’s Modified Eagle’s Medium (DMEM) (Lonza, Verviers, Belgium) with an added 2 mM of L-glutamine, 0.1 mM of non-essential amino acids (NEAA) (Lonza), penicillin/streptomycin antibiotics (100 U/ml) (Lonza) and 10% fetal bovine serum (FBS) (Gibco). The Hs578T cell line was cultured using RPMI-1640 medium (Lonza) with the same supplements added as with the DMEM.

### Constructs and lentiviral transduction

Lentiviral particles were generated for shRNA constructs from the TRC1 library (Sigma-Aldrich, St. Louis, Missouri, U.S.) targeting *PPFIA1* and *VIM*. Constructs for *PPFIA1* were TRCN0000342514, TRCN0000380944, TRCN0000002969, and TRCN0000380097, named as shPPFIA1_14, shPPFIA1_44, shPPFIA1_69 and shPPFIA1_97, and for VIM they were TRCN0000029119 and TRCN0000029121 (Sigma-Aldrich, St. Louis, Missouri, U.S.). The shRNA was cloned into the pLKO.1 vector with the puromycin resistance gene. Lentiviral particles carrying the shRNA constructs for genes of interest and shScramble (shScr) used as a control were generated at the Biomedicum Functional Genomics Unit (FuGU, University of Helsinki, Finland). Transduction of each cell line was performed as briefly described: cells were counted at 100,000 cells/ml, seeded into 12-well plates, incubated overnight, and infected with the lentiviral particles. Selection for the infected shRNA cells was done by using 1 μg/ml puromycin (Sigma-Aldrich).

### Antibodies

Primary antibodies used for immunofluorescence or western blotting were mouse monoclonal α-tubulin (Sigma-Aldrich, St. Louis, Missouri, U.S), mouse monoclonal vinculin (Sigma-Aldrich, St. Louis, Missouri, U.S.), rabbit polyclonal liprin-α1 (Proteintech, Manchester, U.K.), mouse monoclonal CD82 (Abcam, Cambridge, U.K.), rabbit monoclonal sortilin-related receptor 1 (SORL1) (Cell Signaling, Danvers, Massachusetts, U.S.), mouse monoclonal vimentin (Sigma-Aldrich, St. Louis, Missouri, U.S.), mouse monoclonal cytokeratin-13 and rabbit monoclonal cytokeratin-4 (Abcam, Cambridge, U.K.), rabbit polyclonal cytokeratin-13 and rabbit polyclonal cytokeratin-4 (Proteintech, Manchester, U.K.), mouse monoclonal cytokeratin 10 (Invitrogen, Thermo-Fisher Scientific, Rockford, U.S.), goat polyclonal actin (Abcam, Cambridge, U.K.), rabbit monoclonal EEA1 3288 (Cell Signaling), rabbit monoclonal caveolin-1 (D46G3) (Cell Signaling), rabbit polyclonal ZO-1 (Invitrogen). Alexa Fluor phalloidin 488 and 594 (Invitrogen, Thermo-Scientific, Eugene, OR, U.S.) were used to stain the actin cytoskeleton. As secondary antibodies, Alexa Fluor 488 or 594 F(ab’) 2 fragment of goat anti-mouse IgG (H + L) and Alexa Fluor 488 or 594 F(ab’) 2 fragment of goat anti-rabbit IgG (H + L) (Invitrogen, Eugene, OR, U.S.) were used for immunofluorescence microscopy. For immunoblotting, secondary antibodies horseradish peroxidase (HRP) conjugate goat anti-mouse IgG (H + L) (Life Technologies, Rockford, IL, U.S.), HRP conjugate goat anti-rabbit IgG (H + L) (Life Technologies, Rockford, IL, U.S.), and HRP conjugate rabbit anti-goat IgG (Abcam, Cambridge, U.K.) were used.

### Immunoblotting

Cells were grown until they reached 70–80% confluency and lysed using lysis buffer (radioimmunoprecipitation buffer, RIPA) (Sigma-Aldrich, Rockford, IL, U.S.), including protease (complete protease inhibitor cocktail, Roche, Mannheim, Germany) and phosphatase inhibitors (phosphatase inhibitor cocktail, Roche). The lysates were agitated at + 4 °C for 10 min, incubated on ice for 30 min, resuspended gently and centrifuged at 14,000 g after which the supernatants were collected. Protein concentration was measured with an RC DC Protein Assay Kit (BioRad). Loading buffer with 5% β-mercaptoethanol and 2 x Laemmli buffer (BioRad) was added to each sample. Samples were denatured by boiling at + 95 °C for 5 min. For non-reducing conditions β-mercaptoethanol and boiling were excluded. Samples were loaded into the SDS gels (BioRad), from which proteins were transferred to polyvinylidene fluoride (PVDF) membranes (BioRad) using Transfer Blot Turbo (BioRad) equipment. Membranes were incubated at room temperature in a blocking solution consisting of 5% milk powder or 5% bovine serum albumin (BSA) in tris-buffered saline and Tween (TBST) followed by TBST washes. Membranes were incubated with primary antibodies for 1 h at room temperature or overnight at + 4 °C with shaking. After incubation, the membranes were washed with TBST followed by incubation with secondary antibody for 1 h and washing with TBST. Finally, the membranes were incubated with detection reagents (Millipore) for two minutes. Chemiluminescence signals were detected on X-ray films (Kodak) or by using Chemidoc Imaging System (BioRad). Quantification of western blot was measured by ImageLab (BioRad) or by ImageJ, and statistical significance was measured with student’s t-test and error bars as standard deviations. Constructs with efficient knockdown were used in quantification of protein levels.

### Cell culture and immunofluorescence

For three-dimensional (3D) invasive growth assay, cells were grown on type I collagen (2.2 mg/ml, Sigma). Immunofluorescence for two-dimensional (2D) and 3D cultures and quantification of 3D cell cultures were performed as previously described [12, 17]. Alternatively, 40,000 cells/ml were counted and embedded into collagen I drops (Gibco, Life Technologies) in a 24-well plate and incubated for seven days changing medium every day. The cells were fixed using 4% paraformaldehyde in phosphate-buffered saline (PBS), followed by two washes with PBS. Permeabilization was done using 0.25% Triton-X, after which the cells were washed with PBS, incubated with 0.12% glycin, 0.25% Tx-100 in PBS and washed with 0.25% Tx-100 in PBS. After blocking the cells for 60 min in 3% bovine serum albumin (BSA) 0. 25% Tx-100 in PBS or in 15% FBS, 0.3% Tx-100 in PBS (2 h, RT), they were incubated in primary antibody diluted in 1:100 or 1:200 with 3% BSA, 0.25% Triton-X in PBS or in 15% FBS, 0.3% TX-100 in PBS 1 h-overnight at + 4 °C in shaking. The cells were then washed with 0.25–0.45% Triton-X in PBS followed by incubation in a secondary antibody using dilution of 1:400 in 3% BSA, 0.25% Triton-X in PBS or in 15% FBS, 0.3% Tx-100 in PBS for one to four hours with shaking in dark at RT. The cells were washed with 0.25–0.45% Triton-X in PBS minimum three times over several hours and milli-Q water two times with shaking followed by washes with PBS for one hour to overnight. Collagen I drops were mounted in Mowiol containing 1.4-diazabicyclo (2.2.2) octane (DABCO) and 4′6-diamidino-2-phenylindole (DAPI) (Sigma) for nucleus staining.

### Microscopy

Confocal images were taken using a Zeiss Meta 780/880 laser scanning microscope with a Zeiss 40× or a 63×/1.4 N.A. plan-apochromat oil objective, and adjusted and background corrected with ZenLite (2.3 Lite), Adobe Photoshop CS6, Illustrator CS6 software, Corel and/or ImageJ. Brightness and contrast were linearly adjusted using ZEN 2012 (blue edition; Carl Zeiss), Adobe Photoshop CS6 or Photo-Paint X7 (Corel). Single optical sections were used for image display. Three-dimensional collagen contraction images were taken using light microscopy (Leica MZ FLIII). Image editing was done with Adobe Photoshop CS6 and Illustrator CS6 software.

### Co-localization analysis

For co-localization analysis, the confocal images were deconvolved, thresholded by the Costes method and analyzed by the Pearson’s correlation coefficient with Huygens Professional version 18.04 (Scientific, __http://svi.nl__).

### Three-dimensional (3D) cell culture for RNA sequencing

A total of 300,000 cells/ml were counted and mixed with collagen I (Gibco, Life Technologies) diluted in 10 x PBS (Lonza), 1 M NaOH and sterile milliQ water according to manufacturer’s instructions (Gibco, Life Technologies). Collagen I / cell suspension was plated into the 24-well low adhesion plates for the final volume of 500 μl. The collagen I / cell suspension was incubated at + 37 °C for 30 min, after which medium was added on top and changed daily. Cells in collagen I gels were grown for five days after which they were further processed for RNA sequencing. For HNSCC cell lines, the plates were coated with poly(2-hydroxyethyl methacrylate)/poly-HEMA (Sigma-Aldrich) to avoid two-dimensional cell growth and attachment of the cells to the bottom of the plates.

### Collagen contraction and spheroid formation assays

Collagen gel contraction model is a method to estimate cell mediated contracture of the ECM in vitro [18]. MDA-MB-231 control and liprin-α1 knockdown cells were grown in collagen I using the same protocol than in 3D cell culture. Low adhesion 24-well plates were used as cell culture plates. Cell suspensions in collagen I were imaged using optical microscope (Leica MZ FLIII) on days 1, 2, 5 and 6. Relative cell area was calculated using ImageJ software. For spheroid formation assay, 10,000 cells were counted and plated on U bottom 96-well low-adhesion plates and spheroid formation was monitored using light microscope.

### RNA isolation and removal of ribosomal RNA

One milliliter of Trizol (Life Technologies) was added to cell/collagen I mixture which was further mixed with the Precellys ceramic beads followed by homogenization with a homogenisator (Precellys). 0.2 ml of chloroform (Fisher Scientific, Hampton, New Hampshire, US) was added to the samples followed by shaking the samples manually for 15 s. After 2 min incubation in room temperature, the samples were centrifuged at 11,300 rpm for 15 min at + 4 °C. Upper bright phase was kept, 0.5 ml of isopropanol (Fisher Scientific) was added and the solution was mixed. The samples were incubated for 10 min in room temperature and centrifuged at 11,300 rpm for 10 min at + 4 °C after which 1 ml of 75% ethanol (Etax) was added. The samples were mixed and centrifuged at 8800 rpm for 5 min at + 4 °C. Pellets were dissolved to Rnase-free water (Lonza AccuGENE Molecular biology Grade Water) at + 55 °C for 10 min. Isolated RNA was purified using Qiagen RNeasy Mini kit according to Qiagen protocol (Qiagen, Hilden, Germany). Ribosomal RNA was removed by using RiboZero Complete Gold Human kit (Illumina, San Diego, California, US) according to manufacturer’s instructions. Similarly, the second strand cDNA synthesis (NEBNext, Illumina, San Diego, California, US) and purification of double-stranded cDNA by 1.8X Agencourt AMPure XP beads were carried out according to manufacturers’ instructions (Illumina, San Diego, California, US).

### Library preparation

For library preparation, NEBNext Directional RNA library kit was used according to manufacturer’s instructions (Illumina, San Diego, California, US). The PCR amplified library was purified using Agencourt AMPure XP beads according to manufacturer’s instructions (Illumina, San Diego, California, US). The quality of the sequencing library was assessed using Bioanalyzer (Agilent High Sensitivity Chip) and concentrations were measured by Qubit. Library denaturation and dilution were made according to NextSeq 500 system guide (Illumina). Samples were loaded onto the Illumina NextSeq 500 High 75 bp reagent cartridge for RNA sequencing.

### RNA sequencing data analysis

RNA-seq data was pre-processed using QualiMap, FastQC, STAR aligner, and trimmomatic software. Differential expression analysis was performed by DESeq2 package R v3.2.3 and results underwent logarithmic transformation. Statistical significance of the gene expression changes between liprin-α1 knockdown (shPPFIA1) and control cells (shScr) was evaluated by Wald test. Significantly differentially expressed genes were considered those with corrected *p*-values padj < 0.05 (Benjamini-Hochberg). Three different biological replicates per cell line (UT-SCC-42A, UT-SCC-42B, MDA-MB-231) were studied. The RNA sequencing data have been deposited in The *National Center for Biotechnology Information’s* (NCBI’s) Gene Expression Omnibus (GEO, Series accession number GSE108392). In addition, we compared the current RNA sequencing data to our previously published UT-SCC-24B cell line hybridized on Affymetrix GeneChip Human Exon 1.0 ST gene expression microarrays (GEO Series accession number GSE75756) [12]. Genes that were differentially expressed due to liprin-α1 silencing in different conditions and cell lines were compared using Venn diagrams by Venny 2.1 analysis program (http://bioinfogp.cnb.csic.es/tools/venny/index.html). Gene expression data for each sample and condition can be found in Gene Expression Omnibus under accession numbers described above.

### Gene set enrichment analysis (GSEA) data analysis

Gene set enrichment analysis [19, 20] was used to study whether a priori defined set of genes showed statistically significant, analogous differences between control (shScr) and liprin-α1 (shPPFIA1) knockdown cells. The enrichment score (ES) was the result of a gene set enrichment analysis, and reflected how overrepresented the gene set was at the top or bottom of a ranked gene listing. Pathways were ranked by their normalized enrichment score (NES), and were considered significant if their q- and *p*-values were under 0.05. False discovery rate (FDR) signified the probability of a false positive finding and the nominal p-value stated the statistical significance of the ES for the gene set [19, 20].

### Knock-down of CD82 by siRNAs

For knocking-down CD82 in shPPFIA1 HNSCC cells by using a siRNA pool for CD82 (Sigma-Aldrich), UT-SCC-42B cells were counted at 200000/ml, and seeded to six well plates. Cells were grown overnight and siRNA transfection was performed. Transfection reagents used were Lipofectamine RNAiMAX (Thermo-Fisher) and Opti-MEM (Thermo-Fisher). Transfection experiment was performed according to manufacturer’s instructions (Thermo-Fisher). Western blot was performed to verify the efficiency of knock-down. Control cells and CD82 knockdown cells were counted (40,000) and embedded to collagen I (Gibco), and were monitored and incubated for seven days, after which colonies were fixed with 4% PFA and stained with phalloidin. Colonies were quantified by ImageJ and average area of colonies from three replicates/condition was calculated. Error bars were calculated as standard deviation, and statistical significance was measured by unpaired student’s t-test with three different replicates/condition.

## Results

### Liprin-α1 promotes invasive cellular growth in metastatic head and neck squamous cell carcinoma cells

**Fig. 1:**
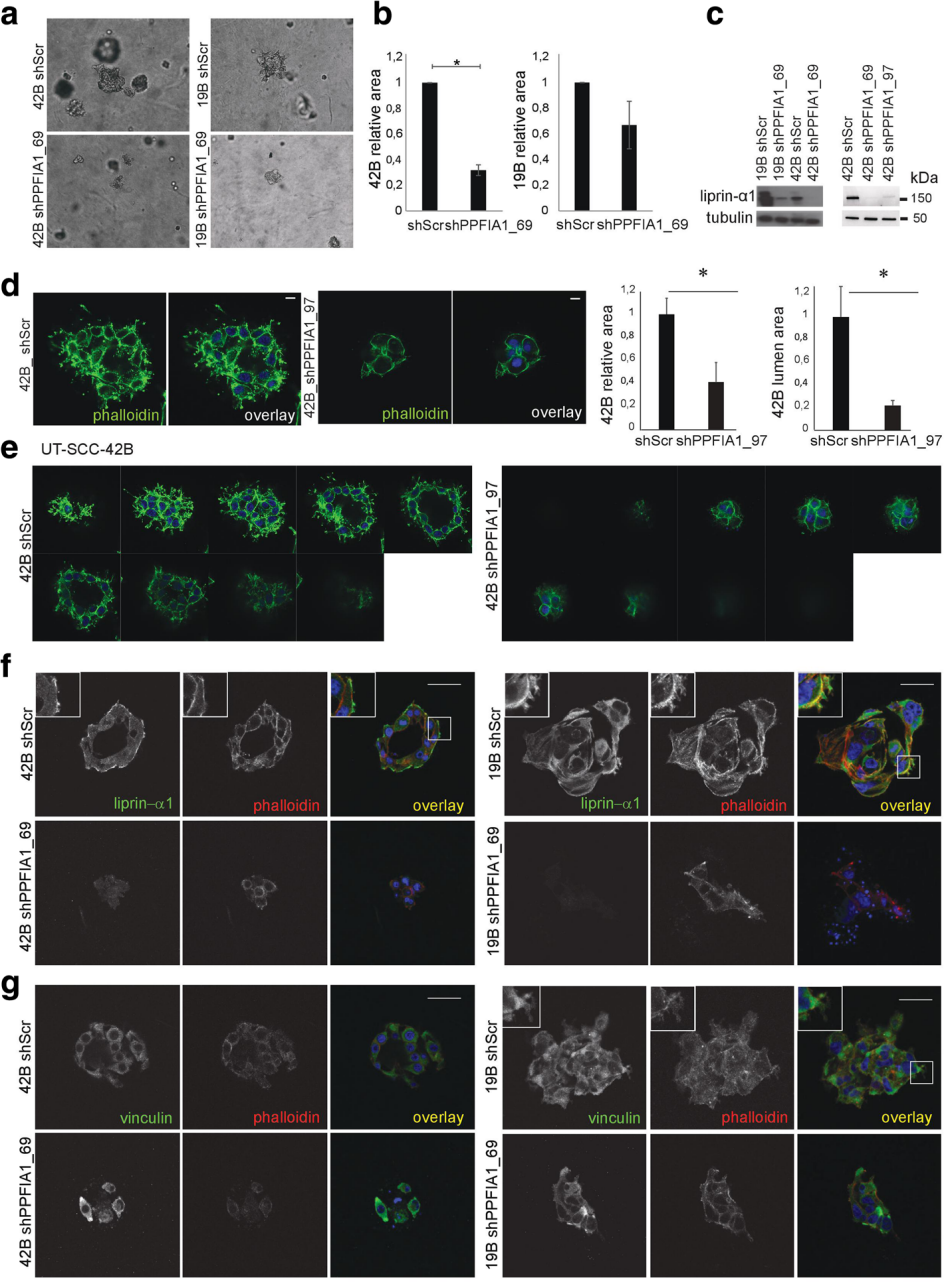
UT-SCC-42B and UT-SCC-19B carcinoma cells were embedded in 3D collagen after shPPFIA1_69 or shScr (control) transduction and cultured for seven days. **a** Representative phase contrast light micrographs showed less cellular outgrowths and invasive phenotype in colonies transduced with shPPFIA1_69 compared to shScr control cells. **b** Quantification of cell invasive growth measured by relative area of the colonies; mean ± SEM; three collagen preparations/stable control (shScr) or knockdown (shPPFIA1_69) cell line. *P < 0.001, unpaired Student’s t-test. **c** Western blot confirmed the efficacy of liprin-α1 knockdown in UT-SCC-19B and UT-SCC-42B cell lines. **d** Quantification of relative colony and lumen area of UT-SCC-42B cells in 3D collagen with shScr and shPPFIA1_97; mean ± SEM; three collagen preparations/stable control (shScr) or knockdown (shPPFIA1_97) cell line. *P < 0.01, unpaired Student’s t-test. Scale bar 10 μm. **e** Lumen formation was diminished in liprin-α1 knockdown cell colonies compared to shScr cells as visualized by Z-stacks. **f** Representative confocal micrographs illustrated liprin-α1 (green) localization in the cytosol and at the cell-extracellular matrix contacts including focal adhesion-like cellular outgrowths. Phalloidin staining (filamentous actin, red) visualizes reduced growth and changes in cytoskeletal elements after liprin-α1 knockdown. **g** Confocal micrographs showed vinculin localization at the cell colonies. Scale bar 50 μm

Based on our previous results showing the function of liprin-α1 in promoting invasive growth of invasive breast cancer cells in 3D collagen environment [12], we explored whether liprin-α1 regulates invasive growth of the highly invasive UT-SCC-42B from metastasis and UT-SCC-19B from primary persistent HNSCC cells as well. UT-SCC-42B cells embedded in 3D collagen I and transduced with shRNA targeting *PPFIA1* reduced cell invasive growth compared to shScr cells, measured as relative growth area of the cells after seven days in culture (Fig. 1a-b). Knockdown of *PPFIA1* led to a less invasive cell phenotype also in UT-SCC19B (Fig. 1a-b), but the relative growth area was not significantly altered as compared to the control cells. The liprin-α1 silencing was also more efficient in UT-SCC-42B compared to the 11q13 positive UT-SCC-19B cell line (Fig. 1c). Liprin-α1 knockdown led to reduced lumen formation, actin cytoskeleton rearrangements and reduced F-actin containing cellular outgrowths in the UT-SCC-42B cell colonies (Fig. 1d-e).

In control cells, liprin-α1 was enriched at the cell colony edge in F-actin and/or vinculin containing structures protruding into the collagen matrix (Fig. 1f-g, Additional file 1: Figure S1). Especially in the lumen-forming UT-SCC-42B cell colonies, liprin-α1 was concentrated to the outer surface of the colony (Fig. 1f, Additional file 1: Figure S1).

### Liprin-α1 knockdown resulted in differential expression of 102 shared genes in HNSCC and breast cancer cells

**Fig. 2:**
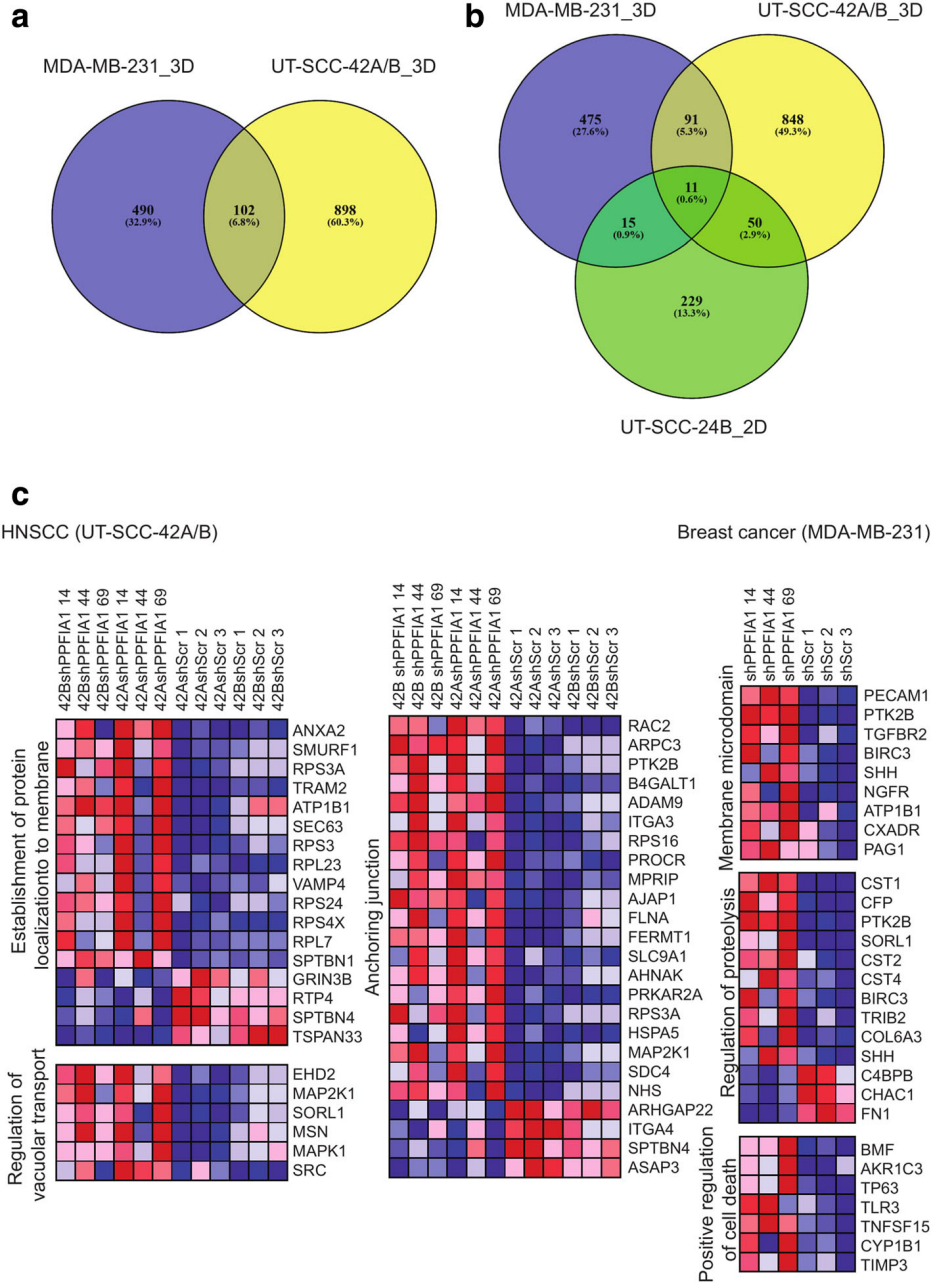
**a** Venn diagram showed a number of shared genes in UT-SCC-42A/B and MDA-MB-231 cell lines grown in 3D collagen I after liprin-α1 silencing. **b** Venn diagram illustrated the number of shared genes in UT-SCC-42A/B and MDA-MB-231 cell lines in 3D collagen I and in UT-SCC-24B cell line grown in 2D cell culture after liprin-α1 silencing. **c** Selected clusters from pathway analysis comparing liprin-α1 knockdown (shPPFIA1) cells to control cells (shScr). Ranking of the clusters was carried out by normalized enrichment scores (NES)

To understand the molecular mechanisms of liprin-α1 in cell adhesion and invasive growth properties, we carried out RNA sequencing to explore gene expression changes after *PPFIA1* knockdown. The metastatic breast cancer cell line MDA-MB-231, laryngeal UT-SCC-42A obtained from primary tumor, and UT-SCC-42B obtained from corresponding neck metastasis were transduced using three different shRNA constructs targeting liprin-α1. In MDA-MB-231 breast cancer cells, there were 592 significantly differentially expressed genes after liprin-α1 knockdown compared to control cells whereas in UT-SCC-42A and UT-SCC-42B cell lines 1745 genes were significantly differentially expressed, when *p*-value was set under 0.05 (Additional file 2: Table S1 and GSE108392). A total of 102 shared genes were differentially expressed in both the breast cancer and HNSCC cell lines, when 592 genes from MDA-MB-231 cell line and 1000 genes from HNSCC data were selected with the most significant *p*-values (Fig. 2a, Additional file 3: Table S2A). To explore the influence of liprin-α1 silencing to gene expression patterns in different cell lines and cell culture platforms (2D vs 3D), microarray and RNA-seq data from HNSCC and breast cancer cell lines were compared (Fig. 2b, GEO Series accession numbers GSE108392 and GSE75756 [12]). A total of 61 differentially expressed genes were shared between two different HNSCC cell lines grown in 2D and 3D conditions (Fig. 2b, Additional file 3: Table S2B), whereas 26 shared genes showed differential expression between shPPFIA1 and shScr samples in HNSCC cells grown in 2D and breast cancer cells in 3D as demonstrated by the Venn diagram (Fig. 2b, Additional file 3: Table S2C). Eleven shared genes showed differential expression between shPPFIA1 and shScr samples in all different conditions as demonstrated by the Venn diagram (Fig. 2b, Additional file 3: Table S2D).

### Gene set enrichment analysis (GSEA) revealed involvement of liprin-α1 in regulation of protein trafficking, cell-cell junctions and membrane microdomains

Next, we studied the effect of liprin-α1 knockdown on the gene expression of specific cellular pathways or gene sets, which better encompasses the overall landscape of the effect of liprin-α1 in the regulation of cell signaling events in cancer cells. GSEA was performed to detect sensitively the overall regulatory patterns derived from the RNA sequencing data (Fig. 2c, Table 1, Additional file 4: Table S3). Knockdown of liprin-α1 in HNSCC cells led to changes in regulation of cell substrate junctions, anchoring junctions (including *ASAP3*), regulation of vacuolar transport (including *SORL1*), phosphatase complex, and establishment or localization of proteins to membrane (including *ANXA2, SMURF1*), or endoplasmic reticulum. In breast cancer cells, GSEA data analysis revealed liprin-α1 in the regulation of membrane microdomains (including *TGFRB2*), regulation of proteolysis (including *SORL1, FN1*) and peptidase activity and regulation of morphogenesis of epithelium. In addition, knockdown of liprin-α1 had a negative effect on peptidase and hydrolase activity, and positive effect on regulation of cell death (including *TP63*) (Fig. 2c and Table 1). GSEA data suggests that liprin-α1 has several crucial functions related to regulation of cellular signaling events and protein trafficking in cancer cells, which are related to the cell-cell or cell-substrate junctions as well as to the composition of the cell membrane. To strengthen the role of liprin-α1 in the junctional and cytoskeletal signaling, collagen contraction analysis was performed with breast cancer cells. Equal amount of control (shScr) and knockdown (shPPFIA1) cells were counted and cells were allowed to grow on low attachment plates inside collagen I for six days. The relative contraction area of the collagen I gel was measured. Collagen gels with control shScr cells were significantly more contracted compared to collagen gels with shPPFIA1 cells (Additional file 5: Figure S2A-C). In addition, liprin-α1 knockdown cells plated without matrix on a low adhesion 96-well plate, did not form tight spheroids opposite to control cells (Additional file 5: Figure S2D). This data further signifies the role of liprin-α1 in the junctional and cytoskeletal pathways.

**Table 1:** Enriched gene sets in GSEA analyses in HNSCC and breast cancer cell lines after liprin-α1 knockdown cultivated in 3D collagen I

Gene Set: HNSCC	NES	FDR q-value	FWER p-value
GO cell substrate junction	2,49	0.000	0.000
GO establishment of protein localization to endoplasmic reticulum	2,47	0.000	0.000
GO protein localization to endoplasmic reticulum	2,45	0.000	0.000
GO anchoring junction	2,35	0.000	0.000
GO protein targeting to membrane	2,2	0.000	0.001
GO establishment of protein localization to organelle	2,14	0.001	0.011
GO regulation of vacuolar transport	2,09	0.001	0.022
GO phosphatase complex	2,08	0.002	0.025
GO establishment of protein localization to membrane	2,07	0.002	0.039
GENE SET: Breast cancer			
GO negative regulation of hydrolase activity	2,74	0.000	0.000
GO regulation of proteolysis	2,54	0.007	0.006
GO negative regulation of peptidase activity	2,5	0.008	0.010
GO regulation of peptidase activity	2,44	0.008	0.013
GO membrane microdomain	2,42	0.008	0.018
GO positive regulation of cell death	2,33	0.016	0.041
GO morphogenesis of an epithelium	2,32	0.015	0.044

### Liprin-α1 suppresses tetraspanin CD82 expression

**Fig. 3:**
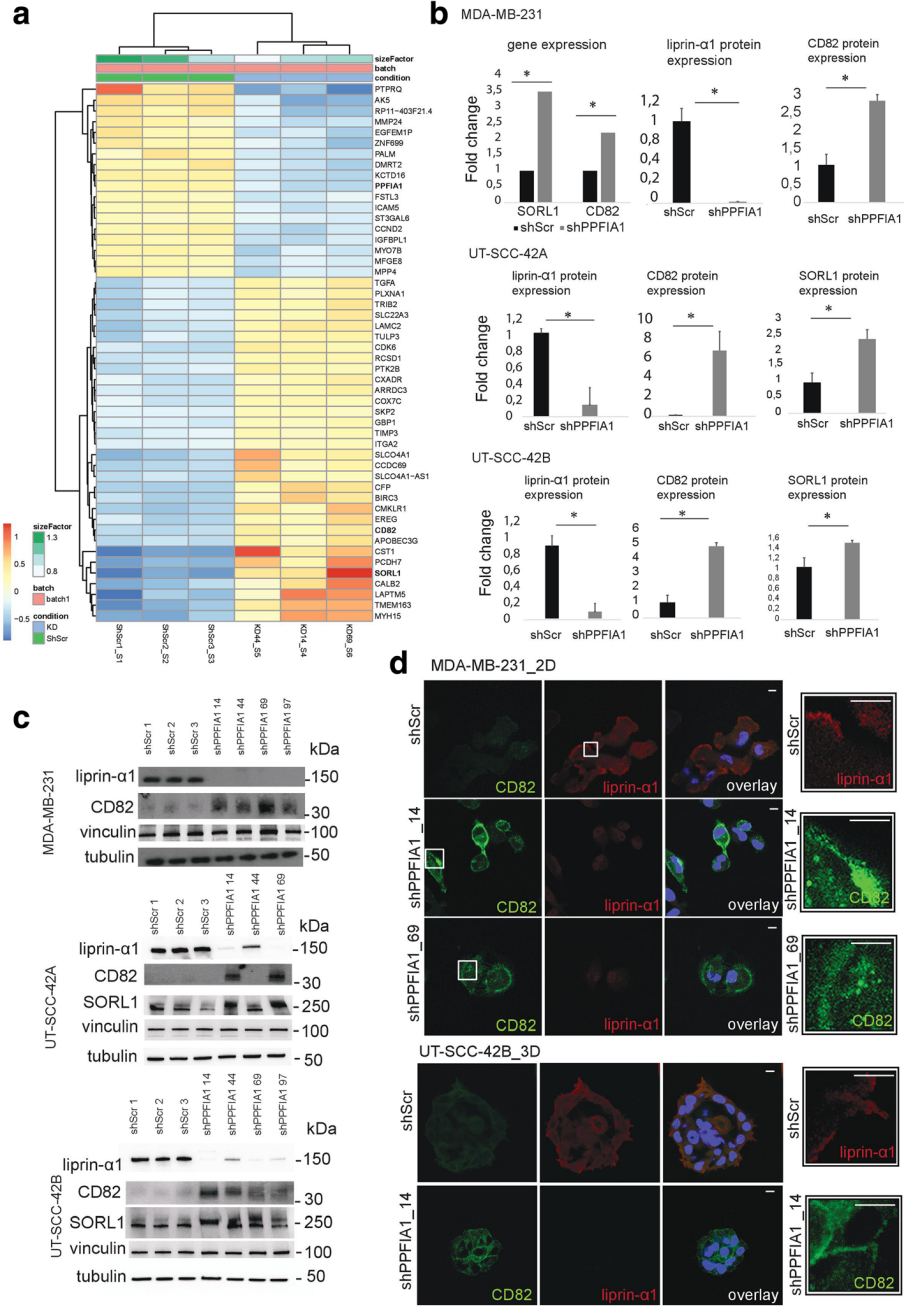
**a** Heat map of top ranked differentially expressed genes in shPPFIA1 vs shScr MDA-MB-231 cells using RNA sequencing. Genes were ranked based on the *p*-value and fold change. Color coding from blue to red depicts gene expression differencies between shPPFIA1 and shScr cells from low to high expression. **b** Gene expression analysis showed upregulation of SORL1 and CD82 at mRNA level in MDA-MB-231 cell lines, and western blot validation showed upregulation of CD82 protein expression in breast and HNSCC cancer cell lines. SORL1 protein expression was increased in HNSCC cells after liprin-α1 knockdown from different biological replicates. Tubulin and vinculin were used as loading controls. Error bar was calculated as standard deviation from different constructs, and value was considered statistically significant when *P < 0,05 calculated as unpaired student’s t-test. **c** Western blot showed the upregulation of CD82 in breast and HNSCC cell line and SORL1 in HNSCC cell lines. **d** Immunofluorescence staining showed upregulation and localization of CD82 to intracellular vesicle-like structures or cell edge in MDA-MB-231 cells after liprin-α1 knockdown. CD82 was at the cell-cell contact sites and near the cell edge in shPPFIA1 UT-SCC-42B cell colonies grown in three-dimensional collagen I. Scale bar is 10 μm and magnification 63×

**Fig. 4:**
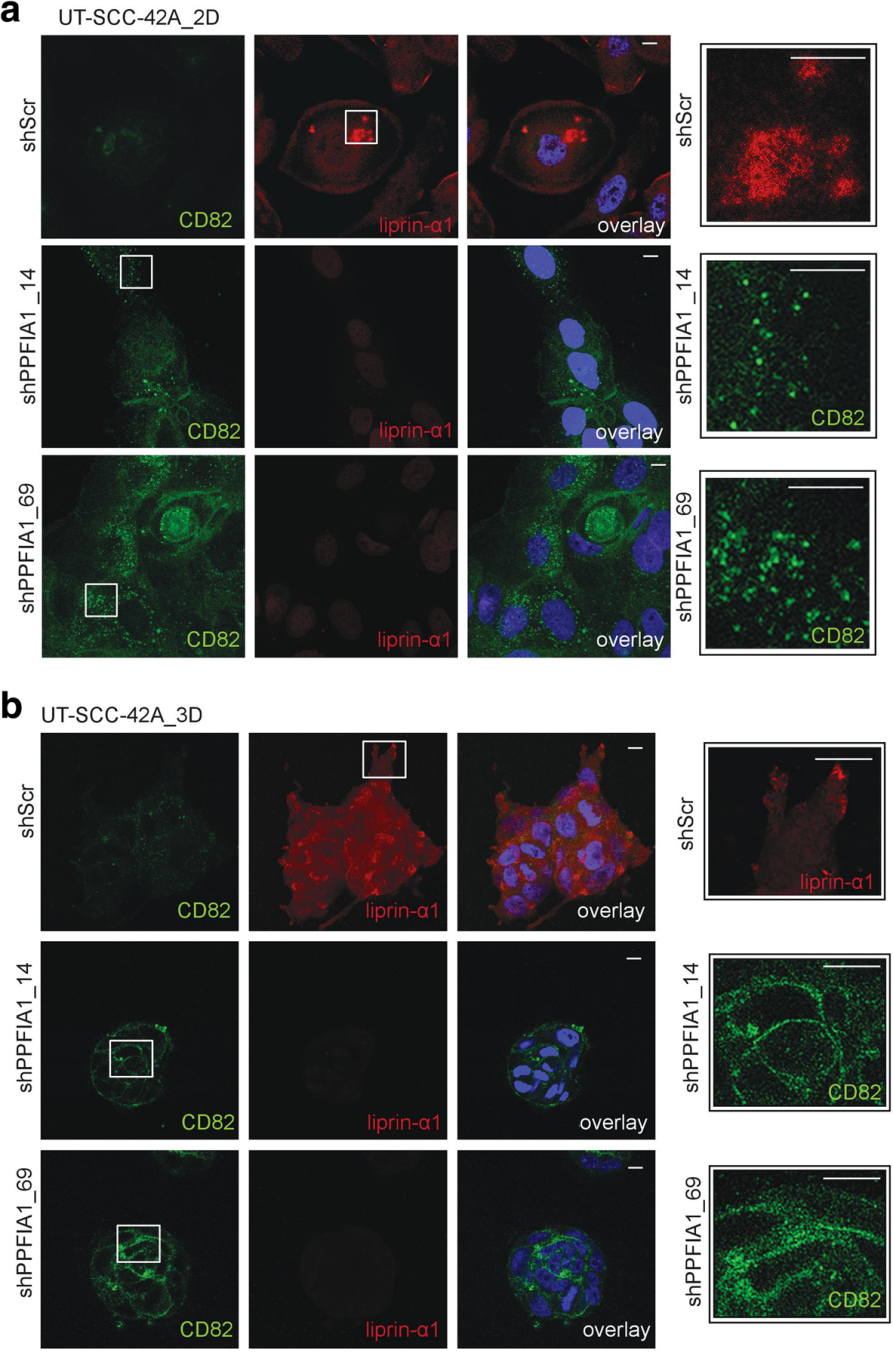
**a** Immunofluorescence staining of CD82 and liprin-α1 in UT-SCC-42A cells cultured in 2D showed localization of CD82 in intracellular vesicle-like structures and at the cell membrane after liprin-α1 knockdown (shPPFIA1). Liprin-α1 was localized in invadosome structures or after the leading edge in shScr control cells. **b** Immunofluorescence staining of CD82 and liprin-α1 in UT-SCC-42A control (shScr) and liprin-α1 knockdown (shPPFIA1) cells in 3D culture. In shScr cell colonies, liprin-α1 was located either as cytosolic or on the edges of the cellular outgrowths in adhesion- or invadosome-like structures. ShPPFIA1 cells showed localization of CD82 mainly at the cell membrane, in cell-cell and in cell-extracellular matrix contacts. Scale bar is 10 μm, and magnification 63×

In the MDA-MB-231 cell line, CD82 was ranked among the top of the upregulated genes in RNA sequencing analysis after liprin-α1 knockdown (Fig. 3a, Additional file 2: Table S1) ((log2) fold change = 1.13, *p*-value = 5.37^-15) and was therefore selected for further studies. Importantly, CD82 was also significantly altered in the HNSCC dataset ((log2) fold change = 1.15, *p*-value = 0.018; Additional file 6: Figure S3A, Additional file 2: Table S1). Interestingly, *SORL1* also showed differences in gene expression after liprin-α1 knockdown in breast cancer and in HNSCC cell lines (Fig. 3a, Additional file 6: Figure S3A, Additional file 2: Table S1) (*SORL1*; MDA-MB-231, *p* = 1.7^-15, (log2) fold change = 1.8, and HNSCC, *p* = 0.0099, (log2) fold change = 1.1). Immunoblotting showed CD82 upregulation in different cell lines knocked down for liprin-α1 (Fig. 3b-c). Interestingly, SORL1 protein expression levels were upregulated in HNSCC cell lines after liprin-α1 knockdown (Fig. 3b-c). In MDA-MB-231 cell line, CD82 was localized either in vesicle-like structures or at the cell edge/membrane as detected in dividing cells after liprin-α1 knockdown (Fig. 3d). Immunofluorescence from UT-SCC-42A cells cultured in 2D revealed that cells with liprin-α1 knockdown contained CD82 positive vesicle-like structures or alternatively, CD82 was localized partly at the cell membrane, while liprin-α1 was detected in invadosome structures in shScr control cells (Fig. 4a, Additional file 6: Figure S3B). CD82 co-localized partly, but not prominently, with caveolin-1 positive vesicles, whereas CD82 did not co-localize with early endosome marker EEA1 (Additional file 7: Figure S4). CD82 was neither co-localizing at the invadosomes, because it was found at different z-stack planes as compared to F-actin positive invadosome cores (Additional file 7: Figure S4). When studying localization of CD82 in 3D cell culture, it was localized at the cell membrane and cell-cell junctions in liprin-α1 knockdown cells (Fig. 4b). Localization to cell-cell contacts was verified with co-localization of F-actin and zona occludens-1 (ZO-1) positive structures (Additional file 8: Figure S5). Liprin-α1 was detected either cytosolic or near the cell edge in adhesion or invadosome-like structures as illustrated in the shScr control UT-SCC-42A cells (Fig. 4b). Downregulation of CD82 by siRNAs partially enhanced invasive capabilities of shPPFIA1_97 UT-SCC-42B cells in 3D collagen I (Additional file 9: Figure S6).

### Modulation of cytoskeletal elements by liprin-α1 in cancer cells

Our previous results from metastatic UT-SCC-24B HNSCC cell line grown in 2D cell culture [12] showed vimentin upregulation in shPPFIA1 versus shScr cells. In the present study, HNSCC cell lines cultured in 3D matrix *VIM* was upregulated in both the RNA ((log2) fold change = 1.7, *p* = 0.0033, Additional file 6: Figure S3A, Additional file 2: Table S1) and protein level (Additional file 10: Figure S7A) in shPPFIA1 cells, which is in conjunction with our previous results. Our previous findings by microarrays showed liprin-α1 knockdown to repress keratin intermediate filaments in metastatic UT-SCC-24B cell line [12], which suggests that keratin network compensates low endogenous vimentin expression in certain types of HNSCC (Additional file 10: Figure S7B-C). Next, we evaluated whether vimentin knockdown affects liprin-α1 expression or localization. After knocking down vimentin in MDA-MB-231 and Hs578T breast cancer cells, which highly express endogenous vimentin, liprin-α1 was not localized near the cell edge or after the leading edge as in control cells, although there were no significant changes at the protein expression levels in these breast cancer cell lines (Additional file 10: Figure S7D-E). Vimentin knockdown had effect on focal adhesion localization exemplified by cytoplasmic staining of vinculin after vimentin knockdown, and lack of liprin-α1 localization at the vicinity of focal adhesions (Additional file 10: Figure S7E). Vimentin knockdown in these cells was not compensated by upregulating keratin intermediate filaments. On the contrary, keratin 13 expression was decreasing after knockdown of vimentin in breast cancer cell lines (Additional file 10: Figure S7D). These results support our previous data on the effect of liprin-α1 knockdown to adhesion and intermediate filament signaling. Furthermore, breast cancer cells with high endogenous vimentin expression were incapable for mesenchymal invasion inside three dimensional collagen I matrix after liprin-α1 knockdown due to disorganized vimentin network [12].

### Significance of PPFIA1 amplification to survival of clinical HNSCC and breast cancer patients

Due to our results showing the association between liprin-α1 and CD82, we explored the significance of *PPFIA1* amplification in survival of clinical HNSCC and breast cancer patients from The Cancer Genome Atlas (TCGA) data. Survival data showed shorter survival for patients with *PPFIA1* amplification when compared to patients with no alteration in both the HNSCC (35.81 versus 64.78 months, *p* = 0.0152) and breast cancer data (125.3 versus 164.3 months, *p* = 0.00998) (cbioportal, TCGA-data, Additional file 11: Figure S8) [21, 22].

## Discussion

*PPFIA1* encoding liprin-α1 shows high correlation between copy number and gene expression in different cancer types and is related to metastatic potential and cell invasive growth in breast carcinoma [1, 11, 12, 23]. The amplification of 11q13 region, where *PPFIA1* is located, is associated to the presence of metastases also in oral squamous cell carcinoma (OSCC) [24]. In the present study, we demonstrated that liprin-α1 enhances invasive potential in metastatic HNSCC cells cultured in 3D collagen I matrix, corroborating our previous findings on the effect of liprin-α1 on cell invasion or invasive growth of motile cells. In this study, we further investigated the mechanism behind the function of liprin-α1 by performing RNA sequencing of breast cancer and HNSCC cells cultured in 3D collagen I environment. When comparing UT-SCC cell lines to metastatic breast cancer after liprin-α1 knockdown, 6.8% of differentially expressed genes were shared between HNSCC and breast cancer, which underlines the heterogeneity of the cancer cell lines. GSEA data analysis demonstrated liprin-α1 involvement in protein trafficking, modulation of membrane composition, as well as cell-cell and cell-substrate signaling. These associations suggest that liprin-α1 is an important player in a variety of different processes related to cell-cell communication and intracellular protein transport in HNSCC and breast cancer cells in 3D environment.

Liprin-α1 knockdown led to upregulation of CD82 cell surface protein, which inhibits formation of invasive structures called microprotrusions during cancer cell invasion [25]. CD82 overexpression correlates with reduced cell growth, migration, invasion and xenograft tumor growth in OSCC [26], and CD82 downregulation is associated with poorer survival in OSCC [27]. Moreover, CD82 has been proposed to decrease cellular dissemination of cancerous cells from primary tumor [28] and invasive potential of different solid tumors (reviewed in [29]). CD82 downregulation associates to increased cell spreading in dendritic cells [30] supporting previous findings on association of liprin-α1 expression to cell edge dynamics and enhanced cell spreading [14]. When we knocked down liprin-α1 in HNSCC and breast cancer cells grown in two-dimensional cell culture, CD82 was located at the vesicle-like structures and showed partial co-localization with caveolin-1. In 3D environment liprin-α1 was localized to adhesion or invadosome-like structures or near cell edge, whereas CD82 was located at the cell edge and cell-cell contacts after liprin-α1 silencing suggesting that the localization of liprin-α1 or CD82 are dependent on environment, matrix availability and cell-cell contacts. Liprin-α1 is a part of β1-integrin signaling and recycling [15, 16], while CD82 modulates β1-integrin maturation and integrin-related cell adhesion in cancer cells [31–33], and is regulated by endocytosis and palmitoylation [34, 35]. In line with this data, caveolin-1, an important scaffolding protein involved in endocytosis and membrane trafficking, is required for CD82 mediated EGFR signaling [36]. Most importantly, we showed in the present work that downregulation of CD82 by siRNAs partially enhanced invasive capabilities of metastatic shPPFIA1 HNSCC cells in 3D collagen providing evidence on the interplay between liprin-α1 and CD82 which is most likely due to the regulative role of liprin-α1 in cell-cell and cell-extracellular matrix contacts.

Gene set enrichment analysis provided several clues for the overall regulatory patterns and genes that are linked to liprin-α1. Interestingly, sortilin-related receptor 1 (*SORL1*), which likely plays a role in endocytosis and sorting and it has association to Alzheimer’s disease [37, 38], was upregulated in liprin-α1 knockdown cells. *SORL1* sorting and quality control occurs in the endoplasmic reticulum (ER)-Golgi complex, and mutations in *SORL1* may prevent trafficking of the protein to the cell membrane due to defects in ER quality control [39]. Interestingly, our gene expression and gene set enrichment analysis data revealed that liprin-α1 is involved in the regulation of the protein signaling related to endoplasmic reticulum. In addition, *ARRDC3,* which is a tumor suppressor and involved in integrin trafficking [40], was upregulated in breast cancer, whereas *FN1* was downregulated after liprin-α1 knockdown. *FN1* has been correlated to poorer prognosis [41] and metastasis formation [42] while a recent publication shows the involvement of liprin-α1 in fibronectin secretion in endothelial cells [16]. In HNSCC, *ASAP3* (alias *ACAP4*) with a potential role in focal adhesions, β1 integrin recycling and migration [43, 44], was downregulated in liprin-α1 knockdown cells. Another gene related to adhesion, integrin inhibition and suppression of focal adhesion formation, *SMURF1* [45], was upregulated in liprin-α1 knockdown cells, which is also likely to have anti-tumor activity [46]. Furthermore, *ANXA2*, whose expression is diminished in dysplastic HNSCC [47] was upregulated in liprin-α1 knockdown cells from HNSCC data.

Our previous data show regulative role of liprin-α1 on vimentin [12] and indeed *VIM* was upregulated in HNSCC cells after liprin-α1 knockdown both in 2D and 3D cell culture platforms. Changes in the proteins related to adhesion and protein recycling underlines the important role of liprin-α1 in cell signaling in 3D environment. Intriguingly, both liprin-α1 and vimentin have important roles in invadosome function in cancer cells [12, 13, 48, 49], vimentin being important in stabilizing and in elongation of mature invadopodia [49]. There is evidence that invadosomes are formed in vivo, and the shape of invadosomes vary depending on the cellular microenvironment [50]. We showed that in 3D environment liprin-α1 localized to adhesion- or invadosome-like structures. Taken together, our GSEA data underlined the role of liprin-α1 in the cell edge functions of cancer cells during cell invasion, shown by modifications in CD82 expression as well as by rearrangements in actin cytoskeleton after liprin-α1 silencing.

Studying the translational modifications following liprin-α1 silencing provided us with better understanding on liprin-α1 association to metastatic progression of the cells. Therefore, we next explored how *PPFIA1* amplification influences the survival of the patients from HNSCC and breast cancer in clinical data. Interestingly, in The Cancer Genome Atlas data *PPFIA1* alteration was identified in 189 (36%) of 528 sequenced head and neck squamous cell carcinoma patients, whereas in breast cancer data set *PPFIA1* amplification was identified in 439 (17%) of 2509 sequenced patients [21, 22]. Survival data showed shorter survival for altered patients when compared to patients with no alteration in both the HNSCC and in breast cancer data [21, 22]. Clinical data emphasizes the importance of *PPFIA1* amplification in cancer cell progression, and as a potential prognostic marker for HNSCC and breast cancer.

## Conclusions

Our study provides novel information regarding liprin-α1 function in cancer cell signaling and strengthens the role of liprin-α1 in regulating cell edge functions during cell invasion. We present novel insight into liprin-α1 dependent regulation of different biological processes and of CD82 which links liprin-α1 to metastatic progression of cancer cells. In conclusion, we show that liprin-α1 controls cell edge protrusions during invasion and metastasis underlining importance of liprin-α1 in cancer cell dissemination and metastatic process of cancer cells.

## Additional files

Additional file 1: **Figure S1.** A: UT-SCC-42B stained with vinculin (green) and liprin-α1 (red) in 3D collagen I matrix. B: Co-localization of F-actin and liprin-α1 in UT-SCC-19B and UT-SCC-42B (left and middle) or vinculin and liprin-α1 in 3D UT-SCC-42B (right) cell colonies. Pearson’s co-localization coefficient and two-channel co-localization maps showing the contribution of each pixel to the co-localization coefficient. Scale bar 30 μm. (JPG 343 kb)

Additional file 2: **Table S1.** Overview of gene expression changes in MDA-MB-231 breast cancer and UT-SCC-42A/B HNSCC cell lines after liprin-α1 knockdown using three different constructs targeting *PPFIA1*. (XLSX 692 kb)

Additional file 3: **Table S2.** A lists of shared genes from RNA-seq data comparisons between shPPFIA1 knockdown and shScr control cells from HNSCC and breast cancer. A: Shared genes between UT-SCC-42A/42B and MDA-MB-231 cells grown in 3D collagen I. B: Shared genes between UT-SCC-42A/42B grown in 3D and UT-SCC-24B cells grown in 2D cell culture. C: Shared genes between MDA-MB-231 grown in 3D and UT-SCC-24B cells grown in 2D cell culture. D: Shared genes in MDA-MB-231 and UT-SCC-42A/42B cells grown in 3D and UT-SCC-24B cells grown in 2D cell culture. (XLSX 22 kb)

Additional file 4: **Table S3.** Overview of the results from the Gene Set Enrichment Data analysis from HNSCC and breast cancer cells line comparing shPPFIA1 knockdown and shScr control cells. (XLSX 491 kb)

Additional file5: **Figure S2.** A: Collagen contraction assay for MDA-MB-231 cell line was performed with shScr control and two different shPPFIA1 constructs. B: The cultures were quantified by relative area of the colony by ImageJ, which showed that control cells displayed better contraction of collagen than knockdown cells. C: Efficiency of liprin-α1 knockdown shown by western blot. D: MDA-MB-231 control cells formed tighter colonies without matrix in a low adhesion 96-well plate compared to the liprin-α1 knockdown cell line. (JPG 139 kb)

Additional file 6: **Figure S3.** A: Downregulation of *PPFIA1* and upregulation of *CD82*, *VIM*, and *SORL1*, which were selected for further studies in UT-SCC cell lines after liprin-α1 knockdown. B: CD82 (green) upregulation and localization to vesicle-like structures and cell edge in UT-SCC-42B cell line after liprin-α1 knockdown shown by immunofluorescence. (JPG 180 kb)

Additional file 7: **Figure S4.** Caveolin-1 and CD82 partly, but not prominently co-localized in UT-SCC-42A cells with liprin-α1 knockdown cells when cultured in 2D. EEA1 and CD82 did not co-localize in the liprin-α1 knockdown cells. Upregulated CD82 localized in different Z-stack plane as compared to phalloidin positive invadosome cores. (JPG 991 kb)

Additional file 8: **Figure S5.** Upregulated CD82 co-localized with phalloidin and ZO-1 in cell-cell contacts after liprin-α1 knockdown in UT-SCC-42A cells in 3D. (JPG 510 kb)

Additional file 9: **Figure S6.** Knockdown of CD82 by siRNAs. A: Western blot verified the partial knockdown of CD82 in UT-SCC-42B shPPFIA1_97 cells compared to shPPFIA1_97 cells transfected with ctrl siRNA. B: Quantification of colony area with three different collagen I gels/condition. Statistical significance was considered to be significant under *P* < 0.05, and error bars were calculated by standard deviations from three replicates. C: Representative images from colonies transfected with either control siRNA or CD82 siRNA. (JPG 270 kb)

Additional file 10: **Figure S7.** A: Vimentin upregulation in UT-SCC-42B cell line was confirmed by immunoblotting using different shRNA constructs for *PPFIA1*. B: Downregulation of keratin 4, 10 and 13 expression levels in insoluble fraction of the UT-SCC-24B cells after liprin-α1 knockdown. C: Keratin 4 (red) and vimentin (green) localization in shScr and shPPFIA1 UT-SCC-24B cells. D: Western blot showed the level of knockdown of vimentin in breast cancer cells. Liprin-α1 protein levels did not alter significantly in control shScr and shVIM cells, but keratin 13 expression was decreased after vimentin knockdown. E: Immunofluorescence images for vimentin (green) and liprin-α1 (red) in MDA-MB-231 and Hs578T breast cancer cell lines and for vinculin (green) and liprin-α1 (red) in Hs578T breast cancer cell line after vimentin knockdown. (JPG 480 kb)

Additional file 11: **Figure S8.** Significance of *PPFIA1* alteration on survival of HNSCC (TCGA, provisional, cBioportal) and breast cancer (TCGA, METABRIC, cBioportal) patients in The Cancer Genome Atlas datasets. (JPG 210 kb)

## Abbreviations

2D: Two-dimensional
3D: Three-dimensional
ATCC: American Type Culture Collection
DABCO: 1,4-diazabicyclo (2.2.2) octane
DAPI: 4′6-diamidino-2-phenylindole
ECM: Extracellular matrix
ER: Endoplasmic reticulum
ERC1: (ELKS/RAB6-interacting/CAST family member 1)
ES: Enrichment score
FDR: False discovery rate
GEO: Gene Expression Omnibus
GSEA: Gene set enrichment analysis
HNSCC: Head and neck squamous cell carcinoma
HRP: Horseradish peroxidase
NES: Normalized enrichment score
OSCC: Oral squamous cell carcinoma
poly-HEMA: Poly(2-hydroxyethyl methacrylate)
PVDF: Polyvinylidene fluoride
RIPA: Radioimmunoprecipitation buffer
shScr: shScramble (small hairpin)
SORL1: Sortilin-related receptor 1
TCGA: The Cancer Genome Atlas
