# Supplementary figures and images for: Liprin-α1 modulates cancer cell signaling by transmembrane protein CD82 in adhesive membrane domains linked to cytoskeleton
Source: Cell Commun Signal. 2018 Jul 13;16:41. doi: 10.1186/s12964-018-0253-y (PMC6045882; doi:10.1186/s12964-018-0253-y)

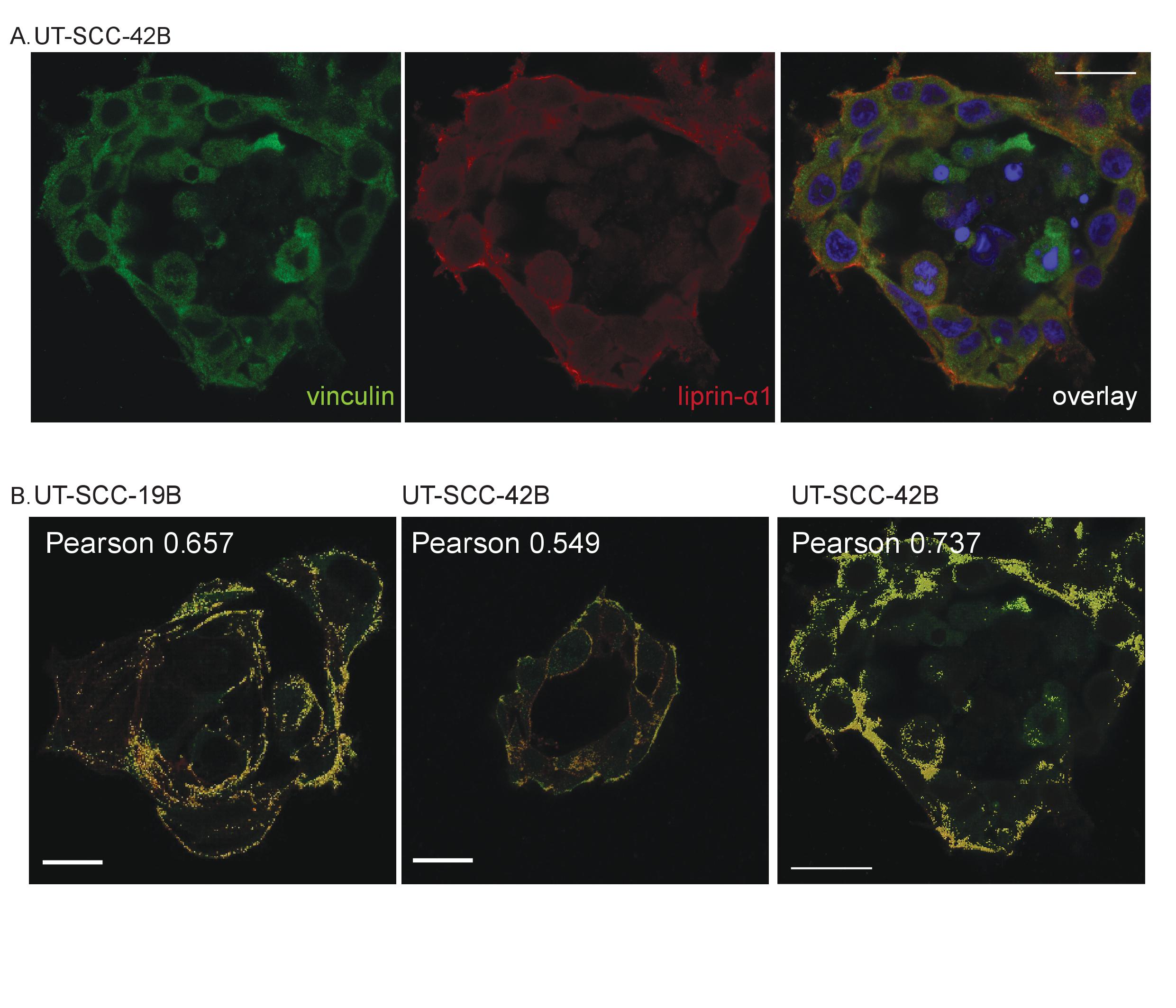

Supplement: Supplementary file 1 — Figure S1. A: UT-SCC-42B stained with vinculin (green) and liprin-α1 (red) in 3D collagen I matrix. B: Co-localization of F-actin and liprin-α1 in UT-SCC-19B and UT-SCC-42B (left and middle) or vinculin and liprin-α1 in 3D UT-SCC-42B (right) cell colonies. Pearson’s co-localization coefficient and two-channel co-localization maps showing the contribution of each pixel to the co-localization coefficient. Scale bar 30 μm. (JPG 343 kb) [file 12964_2018_253_MOESM1_ESM.jpg]

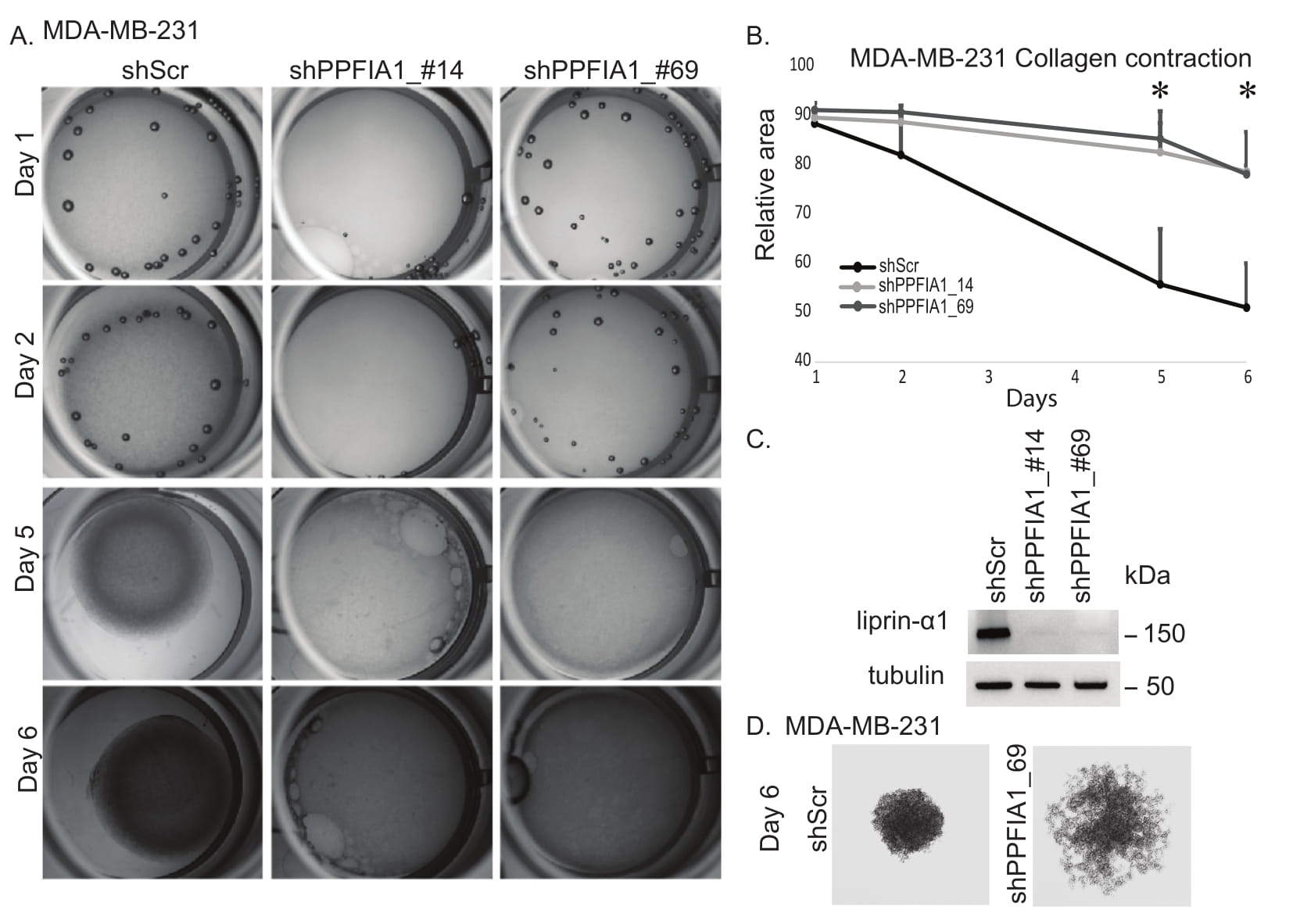

Supplement: Supplementary file 5 — Figure S2. A: Collagen contraction assay for MDA-MB-231 cell line was performed with shScr control and two different shPPFIA1 constructs. B: The cultures were quantified by relative area of the colony by ImageJ, which showed that control cells displayed better contraction of collagen than knockdown cells. C: Efficiency of liprin-α1 knockdown shown by western blot. D: MDA-MB-231 control cells formed tighter colonies without matrix in a low adhesion 96-well plate compared to the liprin-α1 knockdown cell line. (JPG 139 kb) [file 12964_2018_253_MOESM5_ESM.jpg]

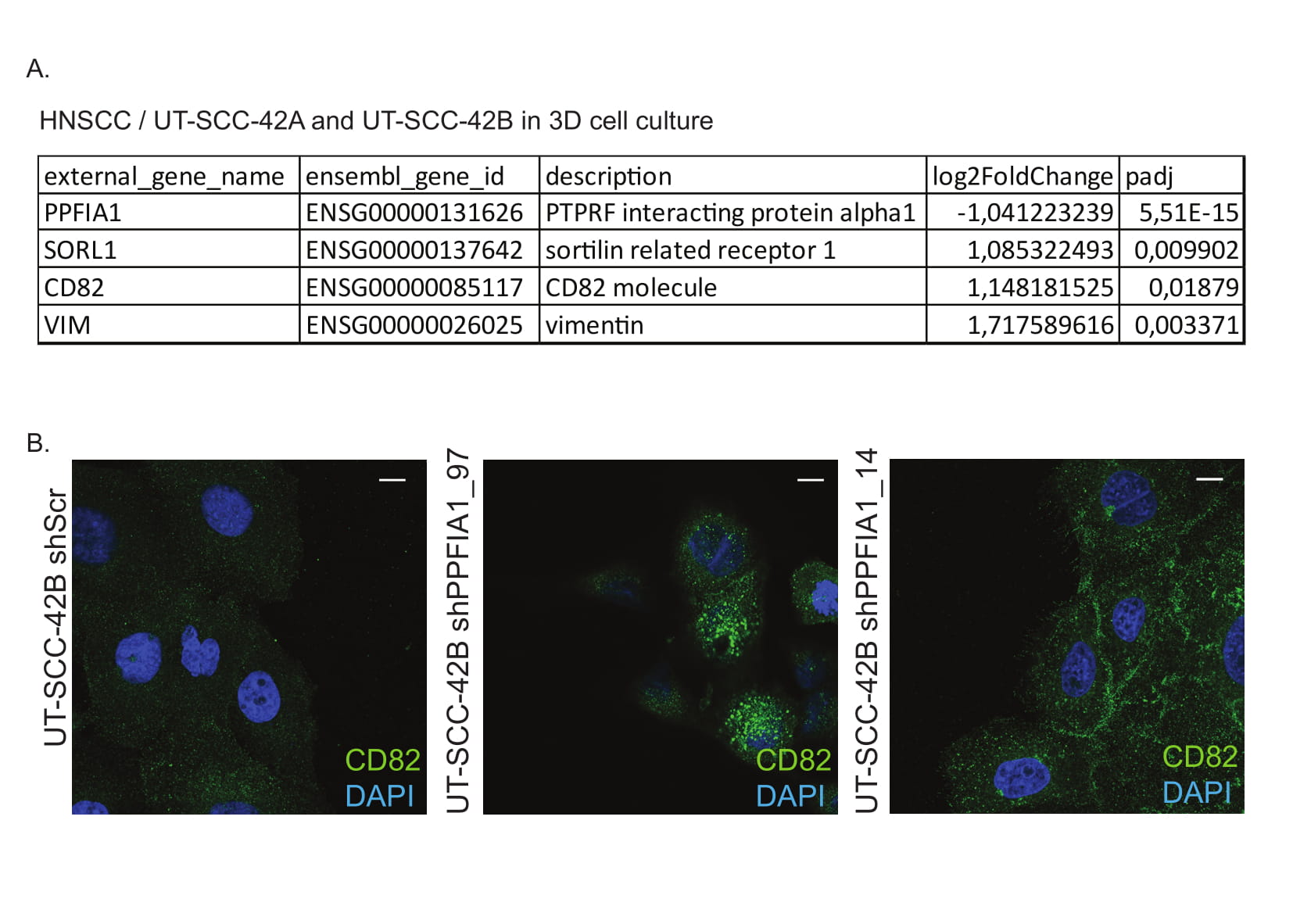

Supplement: Supplementary file 6 — Figure S3. A: Downregulation of PPFIA1 and upregulation of CD82, VIM, and SORL1, which were selected for further studies in UT-SCC cell lines after liprin-α1 knockdown. B: CD82 (green) upregulation and localization to vesicle-like structures and cell edge in UT-SCC-42B cell line after liprin-α1 knockdown shown by immunofluorescence. (JPG 180 kb) [file 12964_2018_253_MOESM6_ESM.jpg]

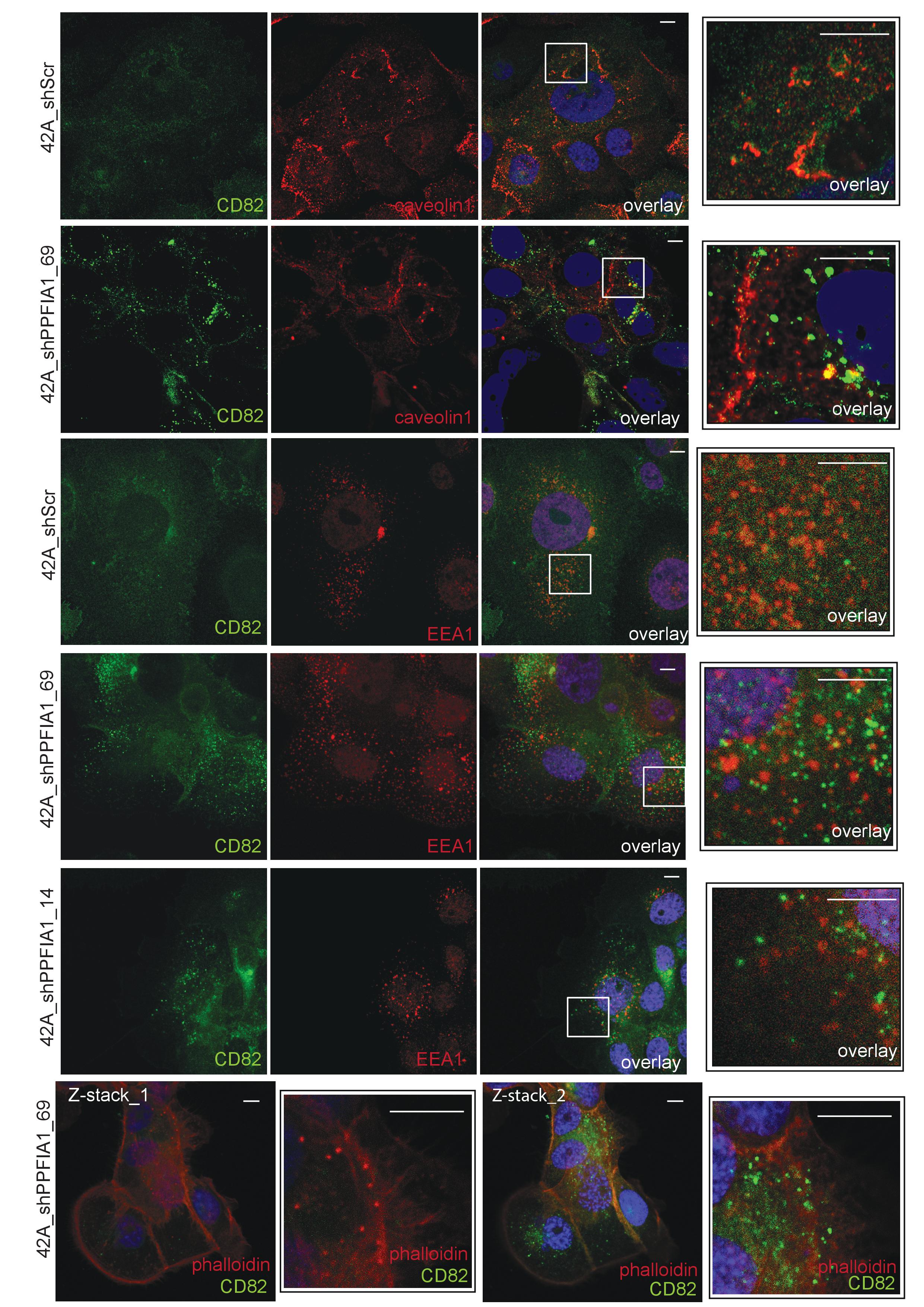

Supplement: Supplementary file 7 — Figure S4. Caveolin-1 and CD82 partly, but not prominently co-localized in UT-SCC-42A cells with liprin-α1 knockdown cells when cultured in 2D. EEA1 and CD82 did not co-localize in the liprin-α1 knockdown cells. Upregulated CD82 localized in different Z-stack plane as compared to phalloidin positive invadosome cores. (JPG 991 kb) [file 12964_2018_253_MOESM7_ESM.jpg]

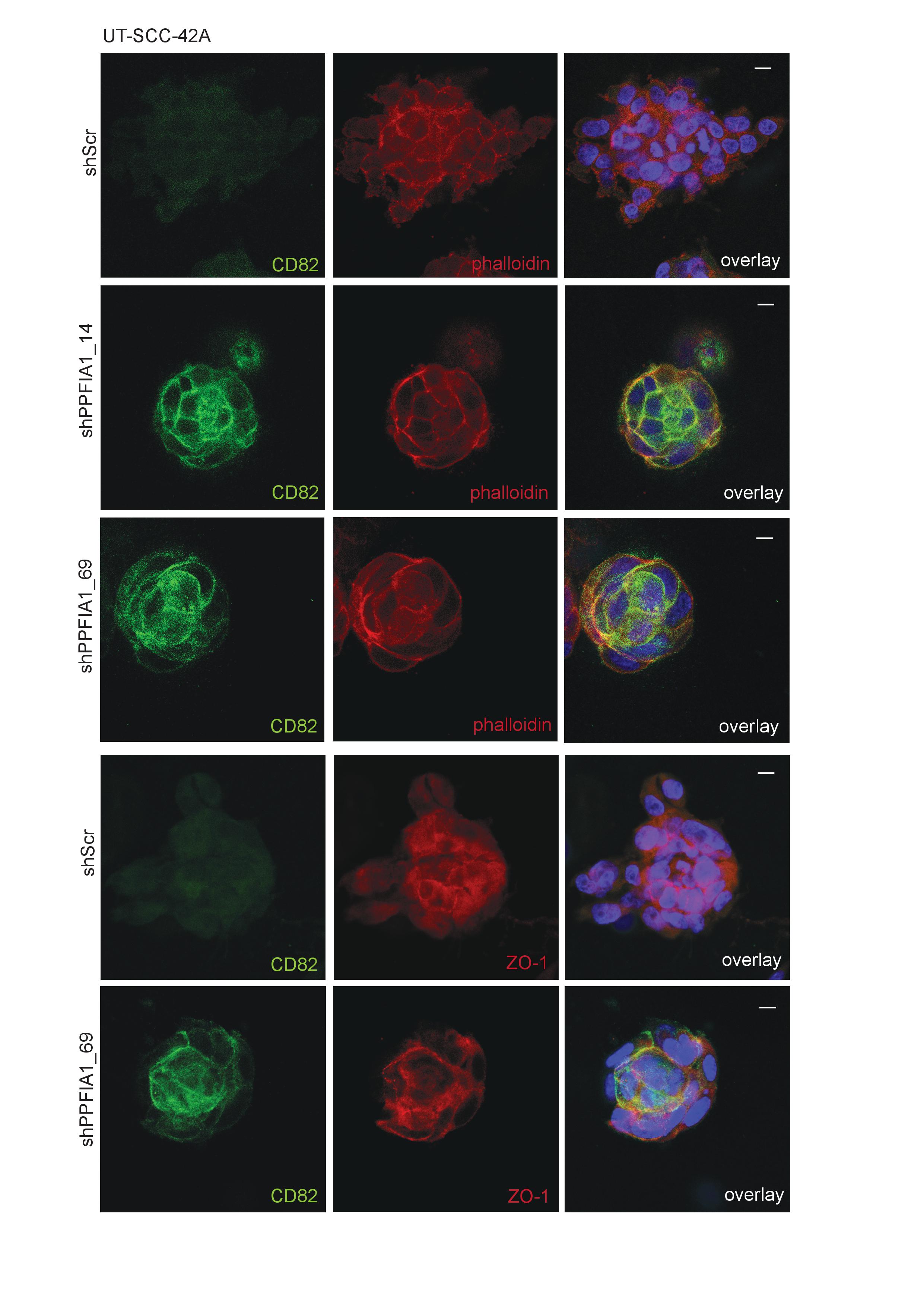

Supplement: Supplementary file 8 — Figure S5. Upregulated CD82 co-localized with phalloidin and ZO-1 in cell-cell contacts after liprin-α1 knockdown in UT-SCC-42A cells in 3D. (JPG 510 kb) [file 12964_2018_253_MOESM8_ESM.jpg]

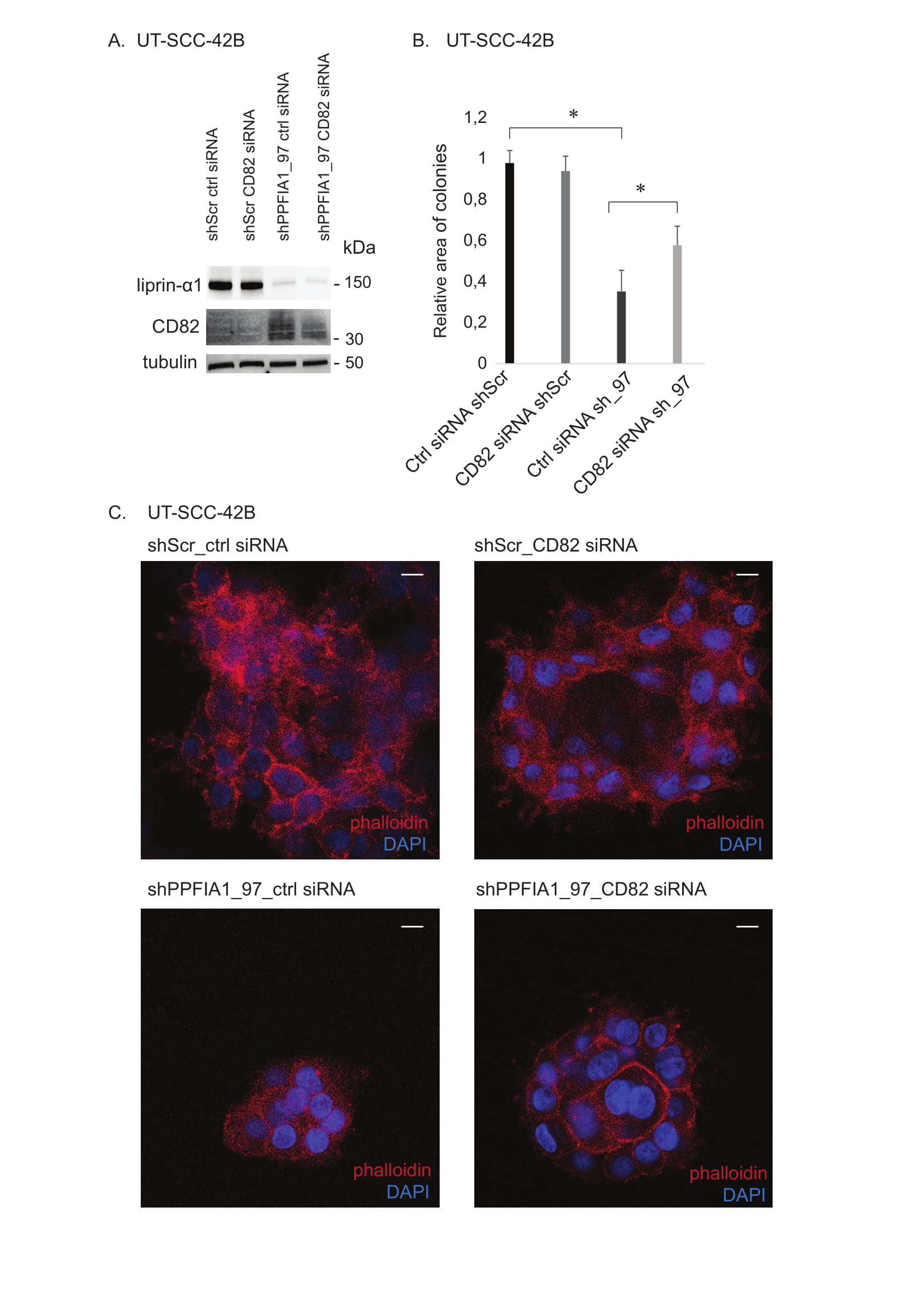

Supplement: Supplementary file 9 — Figure S6. Knockdown of CD82 by siRNAs. A: Western blot verified the partial knockdown of CD82 in UT-SCC-42B shPPFIA1_97 cells compared to shPPFIA1_97 cells transfected with ctrl siRNA. B: Quantification of colony area with three different collagen I gels/condition. Statistical significance was considered to be significant under P < 0.05, and error bars were calculated by standard deviations from three replicates. C: Representative images from colonies transfected with either control siRNA or CD82 siRNA. (JPG 270 kb) [file 12964_2018_253_MOESM9_ESM.jpg]

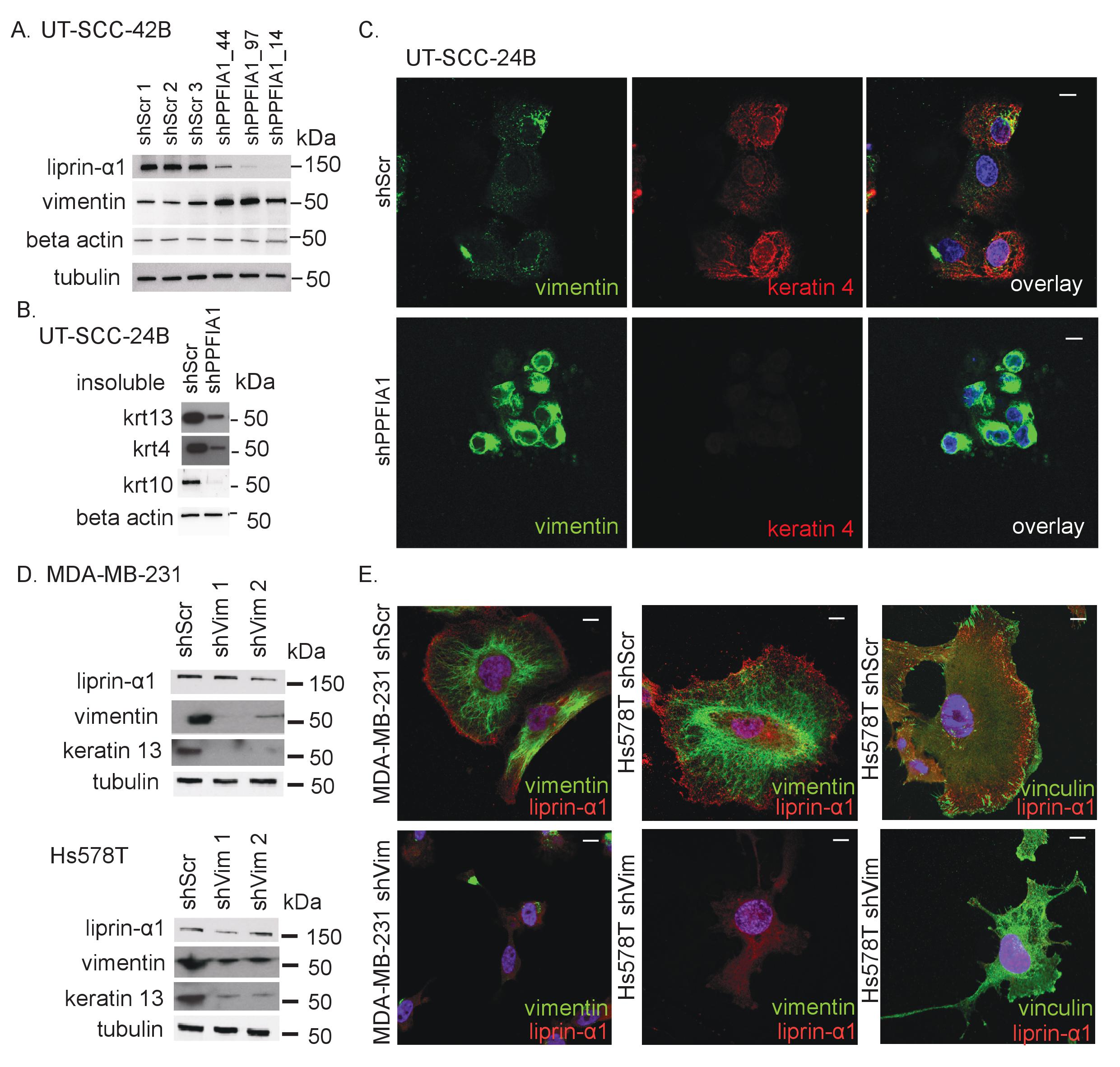

Supplement: Supplementary file 10 — Figure S7. A: Vimentin upregulation in UT-SCC-42B cell line was confirmed by immunoblotting using different shRNA constructs for PPFIA1. B: Downregulation of keratin 4, 10 and 13 expression levels in insoluble fraction of the UT-SCC-24B cells after liprin-α1 knockdown. C: Keratin 4 (red) and vimentin (green) localization in shScr and shPPFIA1 UT-SCC-24B cells. D: Western blot showed the level of knockdown of vimentin in breast cancer cells. Liprin-α1 protein levels did not alter significantly in control shScr and shVIM cells, but keratin 13 expression was decreased after vimentin knockdown. E: Immunofluorescence images for vimentin (green) and liprin-α1 (red) in MDA-MB-231 and Hs578T breast cancer cell lines and for vinculin (green) and liprin-α1 (red) in Hs578T breast cancer cell line after vimentin knockdown. (JPG 480 kb) [file 12964_2018_253_MOESM10_ESM.jpg]

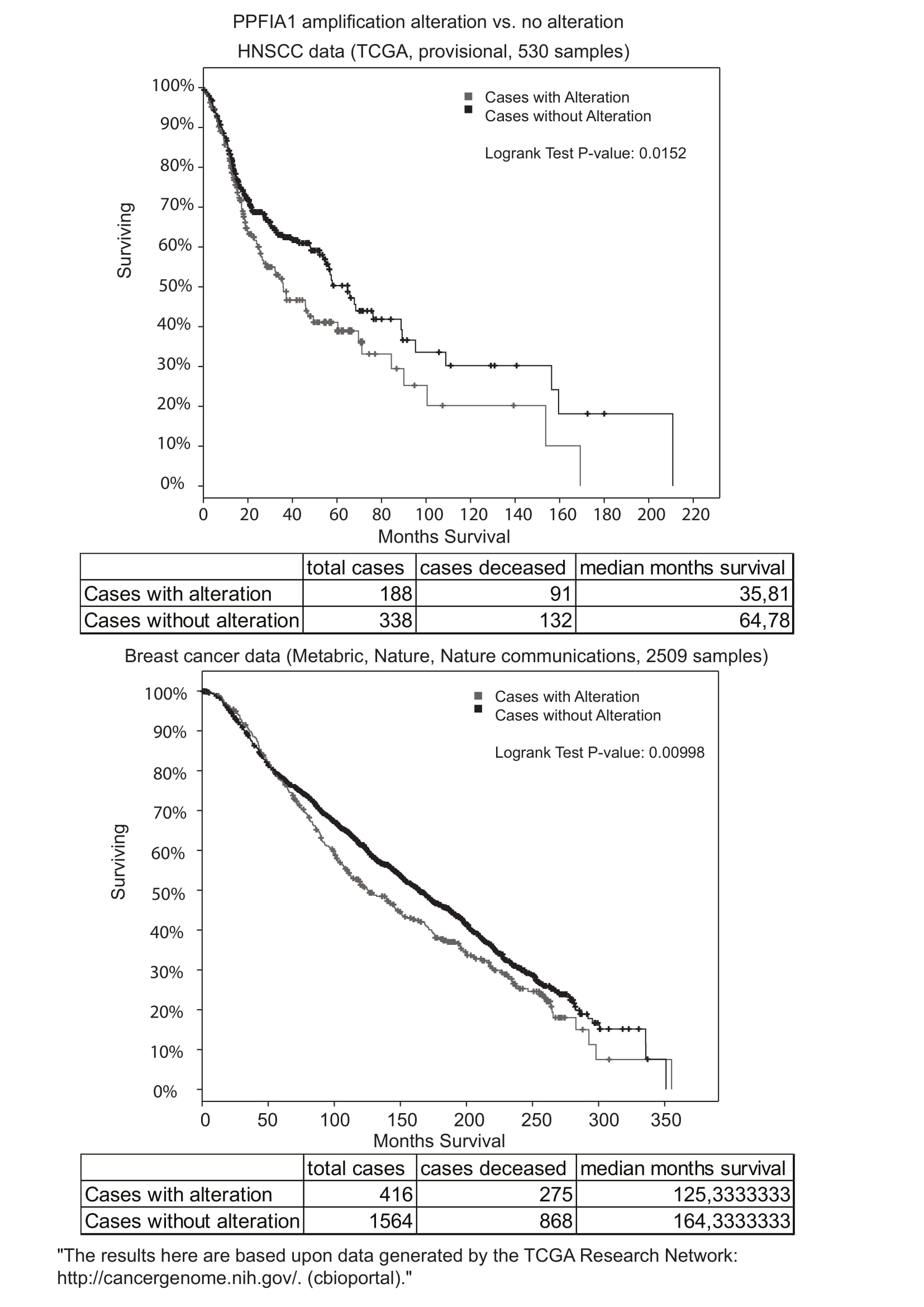

Supplement: Supplementary file 11 — Figure S8. Significance of PPFIA1 alteration on survival of HNSCC (TCGA, provisional, cBioportal) and breast cancer (TCGA, METABRIC, cBioportal) patients in The Cancer Genome Atlas datasets. (JPG 210 kb) [file 12964_2018_253_MOESM11_ESM.jpg]
